# Dentary Morphological Variation in *Clevosaurus brasiliensis* (Rhynchocephalia, Clevosauridae) from the Upper Triassic of Rio Grande do Sul, Brazil

**DOI:** 10.1371/journal.pone.0119307

**Published:** 2015-03-20

**Authors:** Paula Rosario Romo de Vivar Martínez, Marina Bento Soares

**Affiliations:** Laboratório de Paleontologia de Vertebrados, Departamento de Paleontologia e Estratigrafia, Instituto de Geociências, Universidade Federal do Rio Grande do Sul, Porto Alegre, Rio Grande do Sul, Brazil; University of Utah, UNITED STATES

## Abstract

*Clevosaurus* was a cosmopolitan rhynchocephalian genus, known from the Late Triassic to the Early Jurassic. In South America this genus is represented by *C*. *brasiliensis*, an important component of the Linha São Luiz taphocoenosis, on the top of the Norian Santa Maria 2 Sequence of Southern Brazil. The best preserved and most abundant bone elements of *C*. *brasiliensis* are dentaries, in which variations of shape and size are observed. The aim of this study is to describe and evaluate the variation, using geometric morphometrics methods. Geometric morphometric analysis of 10 specimens highlights variations in relative size of the dentary. Most of the variation observed for PC1 (83.3%) is likely related to ontogeny, and PC2 (10.0%) is likely related to taphonomic signatures. The development patterns observed, such as the growth of the dentary, consists of differential growth in length between the posterior portion of the dentary, that grows at a higher rate, regarding the anterior portion of the element. This allometric growth is similar to what is observed in other rhynchocephalians and is accompanied by the allometric skull growth, similar to the trend exhibited by clevosaurs. The taphocoenosis is bimodal (juveniles and adults) with a bias towards adult preservation. Some diagenetic influence is reflected in deformed skulls and this is observed in the tangent-plot. Finally, a strong correlation was detected between the taphonomic signatures and the PC2, regarding specially disarticulation and degree of fragmentation.

## Introduction

Rhynchocephalia, the monophyletic group encompassing *Sphenodon* and its fossil relatives [1], is the sister group of Squamata (lizards, snakes and amphisbaenas) and, together, they comprise the lepidosaurian reptiles (Lepidosauria). Squamata includes more than 9000 extant species, in contrast, the only living genus of Rhynchocephalia is *Sphenodon* from New Zealand [1,2 3]. The current diversity of this group does not reflect its past diversification, when its members were a common component of the continental vertebrate faunas from the Triassic to the Jurassic, with more than 40 fossil taxa. The rhynchocephalians ranged from small to large forms which lived in aquatic and terrestrial ecosystems and experienced different dietary habits such as insectivores, carnivores, omnivores and even herbivores [1,4]

Geometric morphometrics provide an efficient tool for quantitative biology in the study of shape variation and identification of the cause of it [5]. Recently, the use of geometric morphometrics has expanded in vertebrate paleontological studies (e.g. Maxwell and Dececchi [6] in ichthyosaurs; Campione and Evans [7], Hedrick and Dodson [8], Foth and Rauhut [9] in dinosaurs; Fariña and Vízcaino [10], Meloro [11], in mammals).

Concerning Rhynchocephalia, there are only three works in the realm of geometric morphometrics, Jones [2], Meloro and Jones [12], and Humpries and Jones [13]. Before this new approach, ontogenetic studies were carried out using *Sphenodon* and, in fact, some ontogenetic stages are well documented [14,15,16,17]. Regarding fossil Rhynchocephalia, there are some studies with brief notes about ontogeny, as those of Hoffstetter [18], Fraser [19,20], Renesto [21], Reynoso [22], Apesteguía *et al*. [23] and Rauhut *et al*. [4]. However, the only study focusing primarily on the ontogeny of the fossil material was conducted by Reynoso [24], in which were described growth patterns and ontogenetic variation of the jaws and teeth in *Cynosphenodon huizachalensis*. Jones [2] used reconstructions of the following fossil taxa: *Kuehneosarus latus* (outgroup), *Gephyrosaurus bridensis*, *Diphydontosaurus avonis*, *Planocephalosaurus robinsonae*, *Palaeopleurosaurus posidoniae*, *Pleurosaurus goldfussi*, *Brachyrhinodon taylori*, *Clevosaurus hudsoni*, *Clevosaurus bairdi*, *Priosphenodon avelasi*, and 37 specimens of different ontogenetic stages of *Sphenodon*. The author noted that some of the evolutionary trends in fossil rhyncocephalian skulls are very similar to the ones found along the ontogeny of *Sphenodon*, in which the skull undergoes an allometric growth associated with possible by dietary changes. The sample of Meloro and Jones [12], is the same of Jones [2] with the addition of *Clevosaurus brasiliensis*, and the two stem lepidosaurs *Sophieta* and *Marmoretta*. The authors observed the relation between skull shape and dentition, with habitat and dietary preferences. Recently Apesteguía and Carballido [25] in their description of *Priosphenodon minimus* made brief comments on the ontogeny of *Pr*. *minimus*, such as: the differences in size of interpterygoid fenestra between juveniles and adults; the differences of tooth wear, which is stronger in subadults and adults; the form of supratemporal fenestra, which changes from oval to D-shape; the postorbitals, which expand anteroposteriorly during growth; the interpterygoid fenestra and the pterygoid central region narrow during growth; and the nasals overlap the prefrontal and form a hook-like structure in dorsal view that embraces the nasal posterior end, in juveniles specimens [25].

The aim of the current study is, with the help of geometric morphometrics, to understand the morphological variation observed in a sample of 17 specimens of *Clevosaurus brasiliensis* Bonaparte and Sues [26] recovered from the outcrop “Linha São Luiz”, assigned to the top of the Upper Triassic Santa Maria 2 Sequence [27] (Norian), and the potential causes of this variation, whether by ontogenetic processes, sexual dimorphism, taxonomic diversity, or even effect of taphonomy. *C*. *brasiliensis* was the first Rhyncocephalia recovered from the Triassic of South America. More recently, a new taxon, *Sphenotitan leyesi* from the Quebrada del Barro Formation, Northwestern Argentina, also Norian in age, was described [28].

The genus *Clevosaurus* was erected by Swinton (1939) and corresponds to a cosmopolitan genus recorded from the Upper Triassic of Belgium, Brazil, England, and the USA to the Lower Jurassic [29] of China, South Africa and Wales, with a possible occurrence in the Lower Jurassic of Mexico [20, 26, 30, 31, 32, 33]. The clade Clevosaurs is formed by *Brachyrhinodon*, *Polysphenodon* and *Clevosaurus*, and has been recognized in three different phylogenetic analysis: Wu [31], Reynoso [34] and Rauhut *et al*. [4]. According to Reynoso [34] Clevosaurs is supported by the following characters: anterorbital region is less than a quarter of the length of the skull; maxillary teeth with a small medial flange. Bonaparte and Sues [26] formally proposed the family Clevosauridae and defined it as: comprising the last common ancestor of *Brachyrhinodon*, *Polysphenodon* and *Clevosaurus* and all of its descendants [26]. In this sense, Clevosauridae is the formal taxonomic equivalent of the informal grouping Clevosaurs recognized in the phylogenetic analysis aforementioned. The diagnosis of Clevosauridae is as follows: length of anterorbital region one-quarter or less of total skull length; length of lower temporal fenestra more than one-quarter of skull length; anterior (premaxillary) process of maxilla small or absent; maxilla excluded from posterior margin of external naris in *Clevosaurus*; condition uncertain in *Brachyrhinodon*.

The following characters are generally considered diagnostic of *Clevosaurus* by several authors such as Wu [31], Säilä [30], Fraser [20], Fraser and Walkeden [35], Robinson [17], Jones [29, 36] and Apesteguía and Novas [37], although some of them are found in other Rhynchocephalia: a dorsally expanded lateral process of the premaxilla that may exclude the maxilla from the external naris (this was considered an important diagnostic character of *Clevosaurus*, but not exclusive); the premaxillary process of the maxilla is very short or absent; a relatively short snout; a broad contact between the maxillary and jugal; process of the jugal extends posteriorly to meet the squamosal; presence of supratemporal bones; a complete lower temporal bar (although it is often only inferred); the relatively broad orbital portion of the maxilla; suborbital fenestra bounded solely by the lateral process of the ectopterygoid and palatine; a few (<5) large blade-like marginal sub-conical and conical teeth; a reduced number of teeth on the palate; two rows of pterygoid teeth; and the remarkable flanges on the teeth. Thus *Clevosaurus* is still a valid genus based on those characters.

## Material and Methods

### Ethics Statement

Concerning the item Observational and Field Studies, we inform that all fossil materials that were studied by us and presented in this manuscript, have already been collected in field trips between 2000 and 2009 (and housed the Paleovertebrate Collection of at the Universidade Federal do Rio Grande Sul, Brazil). Marina Bento Soares (co-author) is Curator of this collection. Therefore, none authorization was required to access this collection. The access to the other collection cited, FZBRS, was permitted by Dr. Ana Maria Ribeiro, as mentioned in the Acknowledgments.

### Locality and horizon

**Fig 1:**
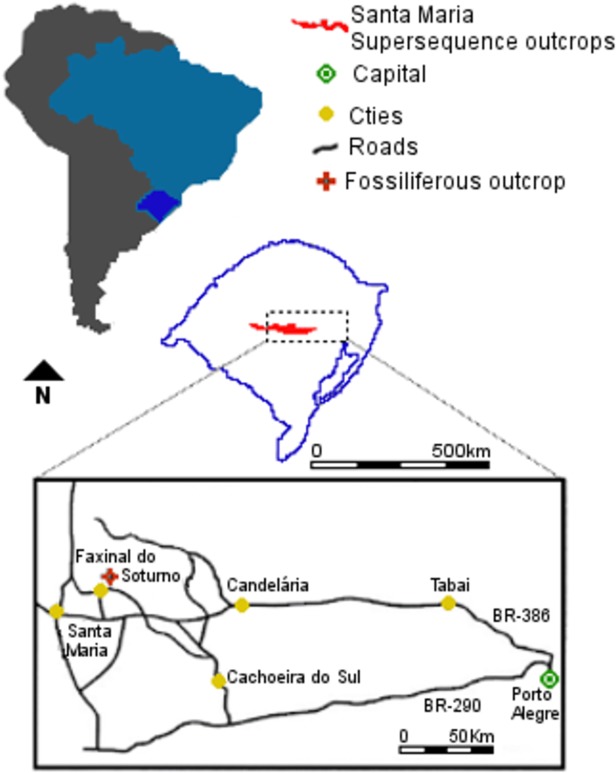
Locality. Location map of the Faxinal do Soturno municipality in the central region of Rio Grande do Sul State, Brazil. Modified from [43].

**Fig 2:**
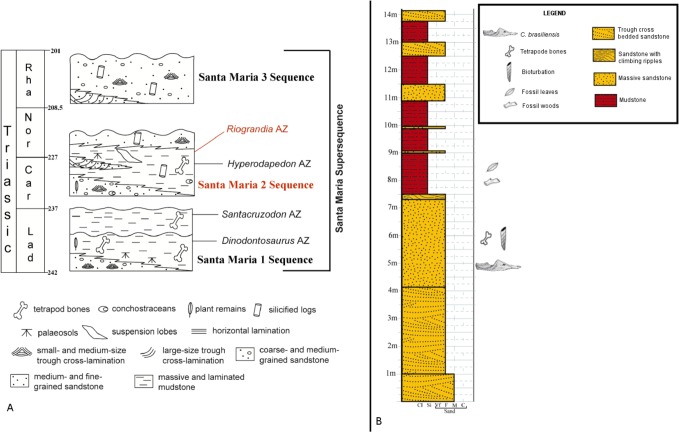
A) Sequence stratigraphy of Brazilian Triassic showing the Santa Maria Supersequence and Riograndia Assemblage Zone modified from [27], with data of [49]. Time scale according to [74] **B)** Stratigraphic column of the outcroup Linha São Luiz, modified from [49] with data of [27]

The material used in this study proceeds from the outcrop Linha São Luiz (29°33ʹ;45”S; 53°26ʹ;48”W), which is located in the Faxinal do Soturno Municipality, Rio Grande do Sul State, Southern Brazil (Fig. 1). This locality is assigned to the top of the Santa Maria 2 Sequence [27] (Norian in age) (Fig. 2A). This sequence includes two systems: a transgressive system tract, composed of red, either laminated or massive mudstone on its base, and a low stand system tract dominantly composed by sandstones, located on its upper portion [27]. This sequence is interpreted as a fluvial/deltaic depositional environment [38].

The Linha São Luiz outcrop assigned to the latter portion, is about 20m thick (Fig. 2B), and it is composed on its base by fine-grained and well selected medium-grained sandstone with cross-bedded, low angle stratification, followed by massive sandstones with cross-bedded stratification The middle portion is composed of mudstones, and the upper portion is composed by rhythmic sandstones and mudstones [39].

The vertebrate fossiliferous levels correspond to the basal portion of the outcrop, where the sandstones are dominant (Fig. 2B). The rhynchocephalian material, referred as *Clevosaurus brasiliensis* in [26], Arantes *et al*. [40], and Arantes (unpublished data), is associated with the non-mammaliaform cynodonts *Riograndia guaibensis*, *Irajatherium hernandezi*, *Brasilodon quadrangularis*, *Brasilitherium riograndensis*, *Minicynodon maieri*, the procolophonid *Soturnia caliodon*, the basal ornithodira *Faxinalipterus minima*, the non-rhynchocephalian lepidosauromorph *Cargninia enigmatica*, and the dinosaur *Guaibasarus candelariensis*, the latter being the largest tetrapod of the assemblage [41,42,43, 44, 45, 46, 47, 48, 49, 50]. The presence of *R*. *guaibensis*, *B*. *riograndensis* and *G*. *candelariensis* allows the correlation of the Linha São Luiz Fauna with other faunas from the top of the Santa Maria 2 Sequence. This faunistic association is individualized as *Riograndia* Assemblage Zone, as proposed by Soares *et al*. [48].

### Specimens

**Fig 3:**
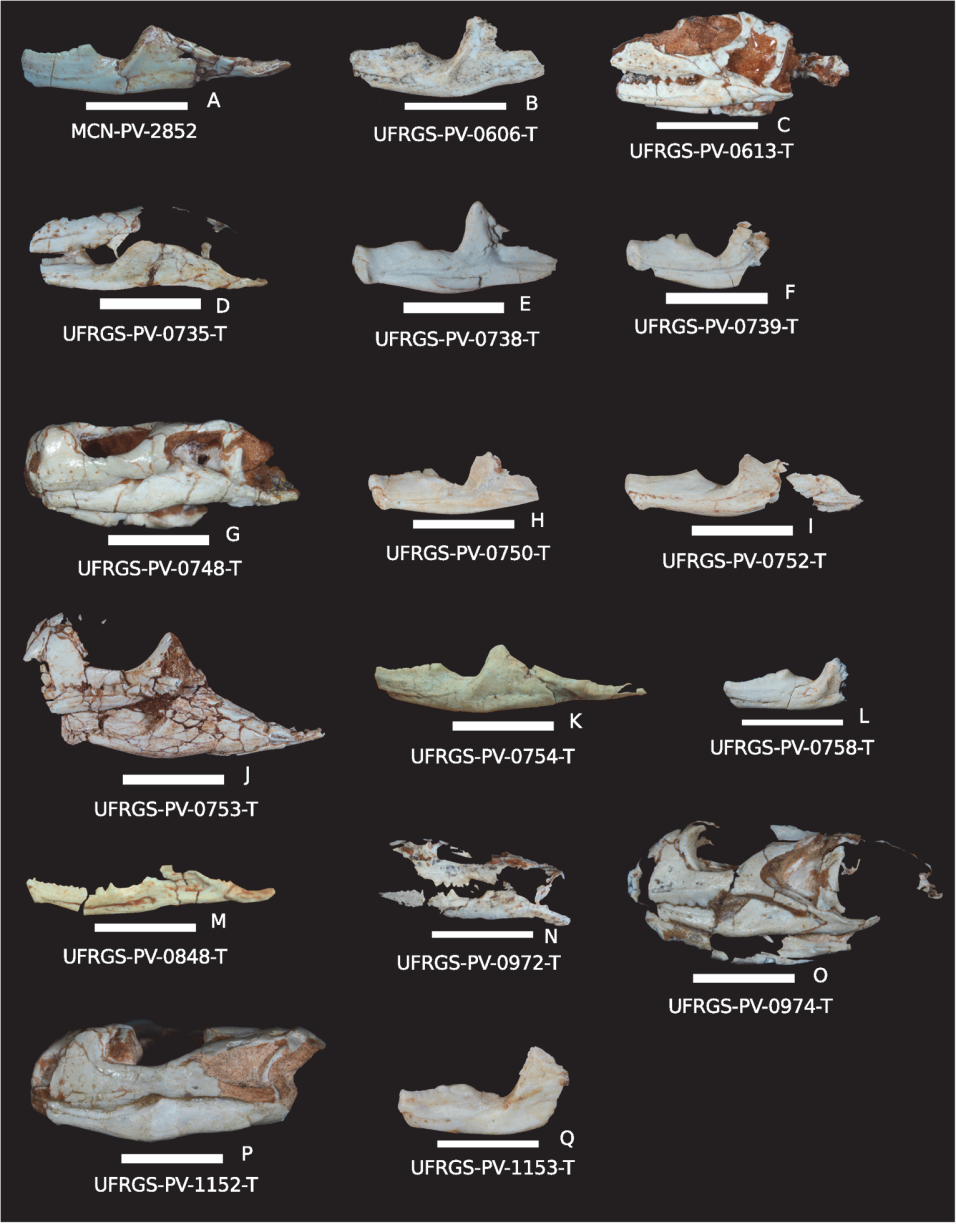
*Clevosaurus brasiliensis* specimens: A) MCN-PV-2852: right lower jaw (mirror-image) in lateral view; B) UFRGS-PV-0606-T: incomplete right dentary in lateral view (mirror-image); C) UFRGS-PV-0613-T: skull with lower jaw in lateral left view; D) UFRGS-PV-0735-T: incomplete skull with right lower jaw (mirror-image) in lateral view; E)UFRGS-PV-0738-T: incomplete right dentary in medial view; F) UFRGS-PV-0739-T: incomplete right dentary in medial view; G)UFRGS-PV-0748-T: skull with lower jaw in lateral left view; H) UFRGS-PV-0750-T: part of the right dentary in medial view; I) UFRGS-PV-0752-T: left lower jaw in lateral view; J) UFRGS-PV-0753-T: skull with lower jaw in lateral left view; K) UFRGS-PV-0754-T: right lower jaw (mirror-image) in lateral view; L) UFRGS-PV-0758-T: incomplete left dentary in lateral view; M) UFRGS-PV-0848-T: left lower jaw in medial view; N) UFRGS-PV-0972-T: skull with lower jaw in lateral left view; O) UFRGS-PV-0974-T: skull articulated with lower jaw in lateral left view; P) UFRGS-PV-1152-T: skull articulated with lower jaw in right lateral view (mirror-image); Q) UFRGS-PV-1153-T: incomplete left dentary in lateral view. Scale bar 1cm

The total sample consists of 17 dentaries attributed to *Clevosaurus brasiliensis* [26, 40], Arantes (unpublished data). These dentaries were found in three different degrees of disarticulation: (1) isolated dentaries (Fig. 3: B, E, F, H, L, Q), (2) dentaries articulated with the angular and the articular complex (surangular, angular and prearticular), but disarticulated from the skull (Fig. 3: A, K, I, M), or (3) complete lower jaw articulated with skull (Fig. 3: C, D, G, J, N, O, P). Some of the specimens are included in the rock matrix and for this reason it was not possible to observe both sides. The specimens used in this study were:

1. Isolated dentaries
  - UFRGS-PV-0606-T: incomplete right dentary (lacking the posterior portion, and a small antero-ventral portion)
  - UFRGS-PV-0738-T: incomplete right dentary (lacking the posterior portion)
  - UFRGS-PV-0739-T incomplete right dentary (lacking the posterior portion, and a small antero-ventral portion)
  - UFRGS-PV-0750-T: part of the right dentary (lacking the posterior portion)
  - UFRGS-PV-0758-T: incomplete left dentary (lacking the posterior portion)
  - UFRGS-PV-1153-T: two unarticulated dentaries (lacking the posterior portion)
2. Dentaries articulated with the angular and the articular complex:
  - MCN-PV-2852: right and left unarticulated lower jaws (associated to the skull but not articulated)
  - UFRGS-PV-0752-T: left lower jaw (lacking the posterior portion)
  - UFRGS-PV-0754-T: right and left unarticulated lower jaws
  - UFRGS-PV-0848-T: left lower jaw
3. Complete lower jaw articulated with skull:
  - UFRGS-PV-0613-T: skull with right and left lower jaws, laterally compressed and lacking the anterior portion
  - UFRGS-PV-0735-T: skull with right lower jaw (lacking the anterior portion)
  - UFRGS-PV-0748-T: skull with right and left lower jaws in occlusion (dorsoventrally compressed)
  - UFRGS-PV-0753-T: skull with lower jaw in occlusion (only the left part preserved)
  - UFRGS-PV-0972-T: skull with right lower jaw (obliquely compressed, lacking the anterior portion)
  - UFRGS-PV-0974-T: skull with right and left lower jaws in occlusion (laterally compressed)
  - UFRGS-PV-1152-T: skull with right and left lower jaws in occlusion (dorsoventrally compressed)

Some of this material has been previously described. The type series of *C*. *brasiliensis* as described by Bonaparte and Sues [26] is composed by the specimens UFRGS-PV-0748-T (holotype) and UFRGS-PV-0613-T (referred specimen). The specimens UFRGS-PV-0735-T; UFRGS-PV-0753-T; UFRGS-PV-0972-T; UFRGS-PV-0974-T; UFRGS-PV-1152-T; UFRGS-PV-1153-T; UFRGS-PV-0972-T were described by Arantes (unpublished data), as *C*. *brasiliensis*. The specimen MCN-PV-2852 was described by Ferigolo [51, 52] but the author did not assign it to *C*. *brasiliensis*, proposing instead that this material could belong to a different taxon. This point of view was rejected by Arantes (unpublished data) who has considered MCN-PV-2852 as *C*. *brasiliensis*, a proposition followed in this study.

Finally, the specimens UFRGS-PV-0606-T; UFRGS-PV-0738-T; UFRGS-PV-0739-T; UFRGS-PV-0750-T; UFRGS-PV-0752-T; UFRGS-PV-0754-T; UFRGS-PV-0758-T; UFRGS-PV-0848-T have not been described yet. In this study, we consider they belong to *Clevosaurus brasiliensis* due to the features they share with this taxon [26] and other *Clevosaurus* (Arantes, unpublished data). These features are: acrodont tooth implantation; additional teeth in the lower jaw, which will decrease gradually and disappear; the evident coronoid process (being half of the height of the lower jaw); absence of a ventral projection in the vertical dentary symphysis with a moderate dorsal development; the posterior process of the dentary expands to the level of the glenoid fossa; sub-conical and conical teeth [20, 26, 37].

#### Dentition

Dentition is a relevant aspect that changes along the ontogeny. Thus, we judge important to make some briefly comments about it. The dentition in *Clevosaurus brasiliensis* is characterized by conical or sub-conical flanged teeth with the long basis mesiodistally directed. This was previously observed by Bonaparte and Sues [26] and Arantes (unpublished data), and this is also a general trend in *Clevosaurus* [20, 30, 35, 36, 53]. According to Bonaparte and Sues [26], in mature *C*. *brasiliensis*, two additional flanged teeth were found in the maxillary followed by one or two smaller, sub-conical teeth. In general we observed this, however, with respect to the additional flanged teeth, in UFRGS-PV-0735-T and UFRGS-PV-1152 we only observed one element; UFRGS-PV-0735-T was eroded and UFRGS-PV-1152 was in occlusion with skull, so it was not possible to observe the teeth. Regarding additional small teeth, we observed between two and four of them in the most posterior portion of the maxilla. In MCN-PV-2852 eight hatchling teeth (these teeth are only observed in medial view) were found. In *C*. *brasiliensis*, between one and three additional teeth are generally found in the dentary and between five and nine hatchling teeth. However, the hatchling teeth tend to be enclosed by the secondary bone in the mature individuals, Table 1.

**Table 1:** Dentition.

Especimen	L. Jaw Add	L. Jaw ht	Mx Add	Mx new Add	Mx ht	Wear Score	Wear General
**MCN-PV-2852**	3	5? ESB	2	2	8?	Meidum	2
**UFRGS-PV-0606-T**	2	? W + ESB				Meidum	3
**UFRGS-PV-0613-T**	2	6	2	3	?W	More	0
**UFRGS-PV-0735-T**	1	? W + ESB	1	3	3?W		3
**UFRGS-PV-0738-T**	1	? ESB					3
**UFRGS-PV-0739-T**	2	5?					2
**UFRGS-PV-0748—T**	?Occ	?Occ	2? Occ	2	?Occ		2.5
**UFRGS-PV-0750-T**	3 or 4	? ESB					2
**UFRGS-PV-0752-T**	3	? W + ESB					2.75
**UFRGS-PV-0753-T**	OCC					Medium	?
**UFRGS-PV-0754-T**	2?	? ESB				Meidium	2
**UFRGS-PV-0758-T**	2	? ESB				Medium	2.25
**UFRGS-PV-0974-T**			2		2?Occ	More	1.75
**UFRGS-PV-0848-T**	2	9?W					1
**UFRGS-PV-0972-T**	2	? W	2	4		More	1
**UFRGS-PV-1152-T**			1? Occ	1?			?
**UFRGS-PV-1153-T**	2 or 3	? W + ESB				Medium	3

L.Jaw Add: number of additional teeth in the lower jaw; L.Jaw ht: number of hatchling teeth in the lower jaw; Mx Add: number of additional teeth in the maxilla; Mx new Add: number of the posterior and small additional teeth in the maxilla; Mx ht: number of hatchling teeth in the maxilla; Wear Score: only in lateral face (Wear Facet). Wear General: observed in the occlusal surface. 1 to 8 = number of present teeth;? plus number = minimal teeth number observed;? = unknown teeth number; Occ: teeth in oclusion; ESB: unknown teeth enclosing by secondary bone.

**Fig 4:**
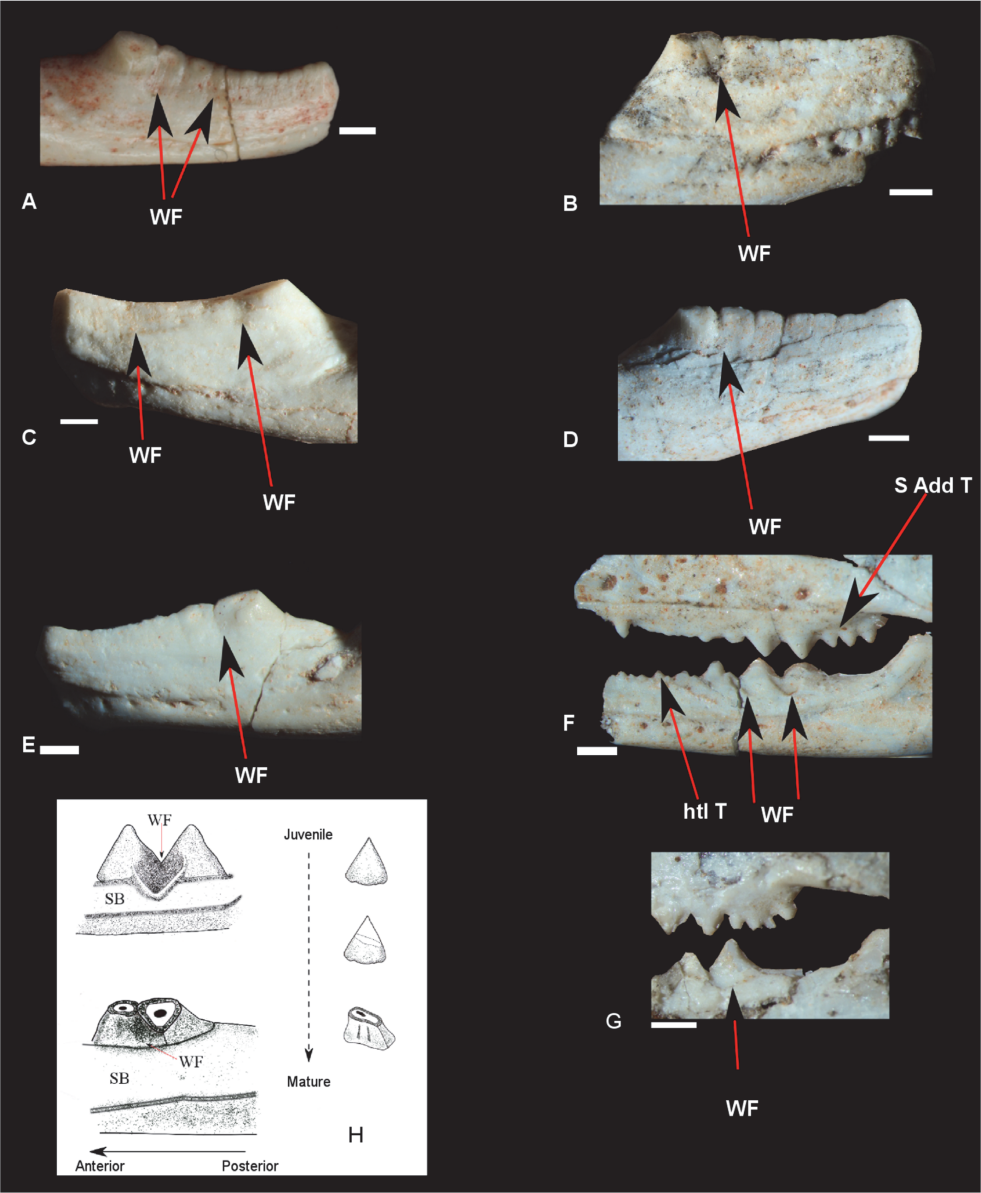
Details of dentition. **A)** MCN-PV-2852; **B)** UFRGS-PV-0606-T; **C)** UFRGS-PV-0752-T; **D)** UFRGS-PV-0754-T; **E)** UFRGS-PV-0758-T; **F)** UFRGS-PV-0613-T; **G)** UFRGS-PV-0972-T; **H)** Scheme of teeth wear along ontogeny. WF: Wear Facet; htl T: hatchling tooth; S Add T: Small additional teeth; SB: secondary bone. Scale bar 0.5cm.

**Fig 5:**
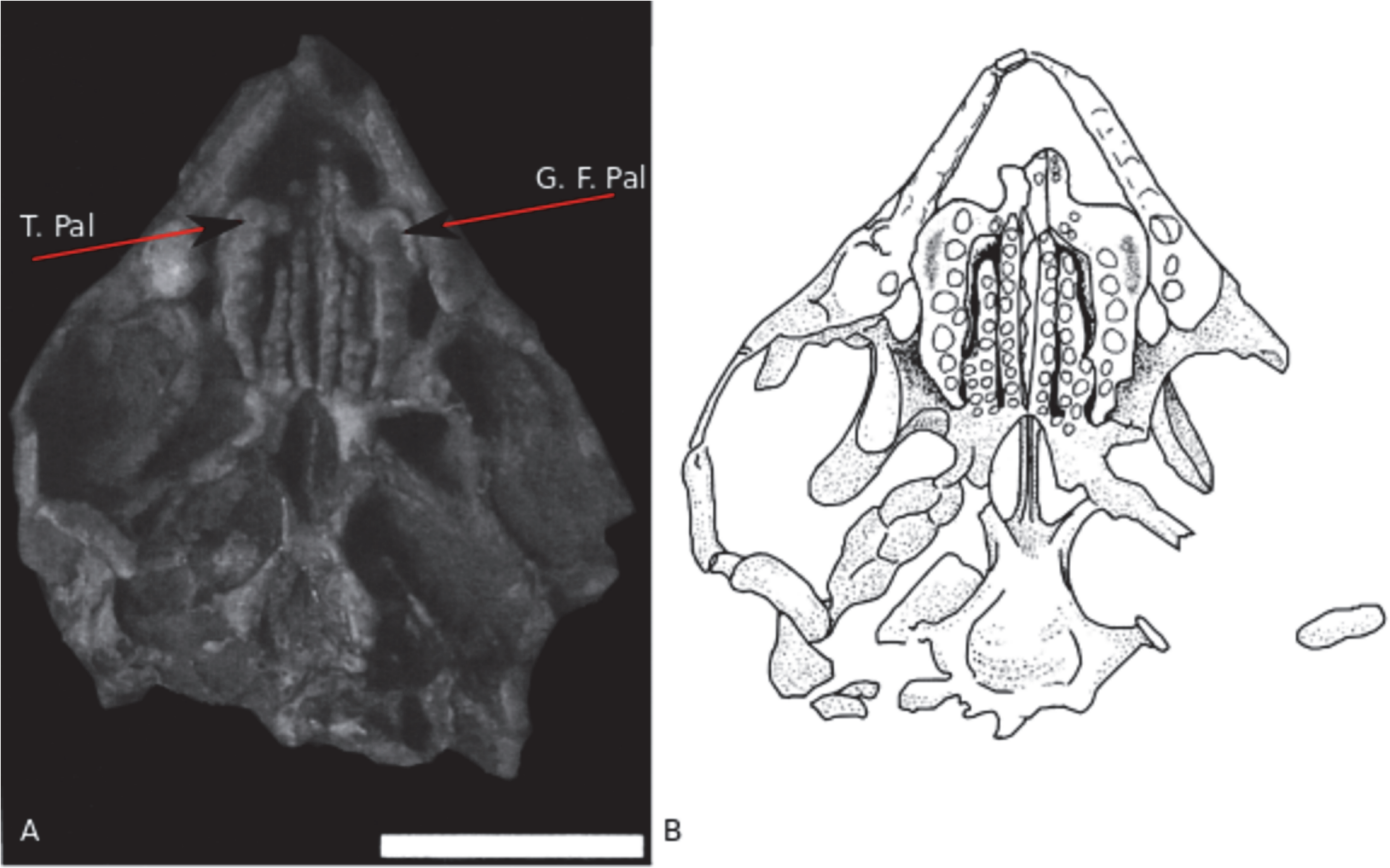
Palatal view of MCN-PV-2852 skull. Scale bar 1 cm. T.Pal: Palatine teeth; G.F.Pal: groove for the external flange of the palatine, modified from [41, 42]

Other features observed in *C*. *brasiliensis* dentition include two types of wearing in the dentary teeth: (1) Triangular score marks on the lateral side of the teeth, produced by the orthal occlusion with the maxillary teeth. These score marks are reported in other *Clevosaurus* as well [20, 30, 35]. This was also previously observed in UFRGS-PV-06013-T by Bonaparte and Sues [26], and it was observed by us in UFRGS-PV-0972-T, and in other specimens (MCN-PV-2852, UFRGS-PV-0606-T; UFRGS-PV-0752-T; UFRGS-PV-0754-T; UFRGS-PV-0758-T), but they comprised smoother marks (Fig. 4); (2) Marks in the occlusal surface of the crown produced by the occlusion with the “groove for the external flange” of the palatine (pers. Obs. PRM) and palatine teeth (Fig. 5) [20] (pers obs. PRM), with the more worn teeth placed in the anterior portion, some of them covered by the secondary bone, forming the edentulate portion of the beak-shaped dentary [20. 35]. This pattern of wearing in the teeth of *Clevosaurus* is well known [20, 30, 35]. We suspect that these distinct patterns of wearing among the individual of our sample are reflecting different ontogentic stages, as already observed by Fraser [20] and Fraser and Walkden [35], and in *Priosphenodon minimus* by Apesteguía and Carballido [25], that could be statistical correlated with the results from PCA, however, other authors mentioned that when acrodont teeth are susceptible to worn, and hence the extant of wear is not thought to correlate simply age or skull size[17, 29].

### Data collection

**Fig 6:**
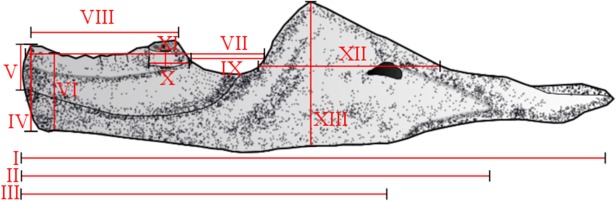
Linear measurements. **I)** lower jaw length; **II)** dentary length; **III)** length from the most anterior and ventral point of the secondary bone to mandibular fossa; **IV)** anterior height of the dentary; **V)** anterior height of the secondary bone; **VI)** height of the dentary in the inflexion point; **VII)** length from the anterior portion of the dentary to anterior portion of the coronoid process; **VIII)** length from the anterior portion of the dentary to the posterior portion of the laster additional tooth. **IX)** length from the anterior portion of the last additional tooth to the anterior portion of the coronoid process; **X)** length of the base of the last additional tooth; **XI)** height of the last additional tooth; **XII)** length from the coronoid process; **XIII)** height of the coronoid process.

Linear measurements of the different structures were taken with a calliper (Fig. 6 and Table 1). Among the specimens that have two lower jaws, we selected the best preserved, in order to reduce possible taphonomic biases. The images were obtained by two different steps: (1) all specimens were photographed in lateral, medial or both views, when it was possible, with a digital camera Canon EOS Rebel T3i using macro lens Ef 35–70mm. Only the specimen MCN-PV-2852 was photographed using a Canon EOS Rebel XSi camera with macro lens Ef 50mm. After this, mirror images were obtained of the right dentaries, in lateral view, and of the left dentaries, in medial view, so that images of all the specimens were oriented in the same direction; (2) since not all dentaries are complete, a search for missing data was made (which will be explained in the next section), using the previously linear measures taken. The estimated values were employed for the reconstruction of the incomplete dentaries (Table 2).

**Table 2:** Linear measurements.

Especimen	I	II	III	IV	V	VI	VII	VIII	IX	X	XI	XII	XIII
**MCN-PV-2852**	**2.710**	**2.097**	**2.065**	**0.355**	**0.226**	**0.323**	**1.100**	**0.746**	**0.355**	**0.194**	**0.129**	**0.839**	**0.677**
**UFRGS-PV-0606-T**	2.497	**1.920**	**1.887**	**0.333**	**0.189**	**0.310**	**1.060**	**0.730**	**0.325**	0.184	**0.094**	0.821	**0.750**
**UFRGS-PV-0613-T**	**1.768**	1.755	1.534	0.210	**0.113**	**0.133**	**0.868**	**0.679**	**0.207**	**0.113**	0.172	**0.604**	**0.468**
**UFRGS-PV-0735-T**	2.380	1.958	1.746	**0.210**	**0.092**	**0.148**	0.924	0.656	**0.358**	**0.203**	0.176	**1.009**	**0.485**
**UFRGS-PV-0738-T**	2.821	2.062	2.172	**0.375**	**0.196**	**0.300**	**1.050**	**0.592**	**0.440**	**0.286**	**0.107**	0.907	**0.880**
**UFRGS-PV-0739-T**	2.457	1.936	1.917	0.282	**0.206**	0.272	**1.048**	**0.605**	**0.320**	**0.206**	**0.168**	0.830	0.645
**UFRGS-PV-0748-T**	**2.580**	**1.910**	**1.776**	**0.208**	0.170	0.226	0.940	0.694	0.320	0.244	0.185	**0.816**	**0.573**
**UFRGS-PV-0750-T**	2.107	1.944	1.804	**0.355**	0.132	**0.305**	**0.965**	**0.668**	**0.323**	**0.145**	**0.091**	0.646	**0.590**
**UFRGS-PV-0752-T**	**2.791**	**2.233**	**2.163**	**0.298**	**0.198**	**0.278**	**1.093**	**0.791**	**0.333**	**0.186**	**0.058**	0.907	**0.620**
**UFRGS-PV-0753-T**	**2.675**	**2.351**	**2.351**	**0.310**	0.174	**0.355**	**1.083**	**0.688**	**0.320**	0.220	0.195	**1.094**	**0.953**
**UFRGS-PV-0754-T**	**2.78**	**2.094**	**2.063**	**0.390**	0.192	**0.300**	**1.094**	**0.780**	**0.344**	**0.200**	**0.100**	**0.781**	**0.680**
**UFRGS-PV-0758-T**	2.388	1.830	1.581	**0.286**	**0.159**	**0.244**	**0.924**	**0.640**	**0.351**	**0.203**	**0.087**	0.819	**0.431**
**UFRGS-PV-0974-T**	**2.167**	**1.705**	**1.639**	**0.243**	0.166	**0.283**	0.921	0.615	0.312	0.176	0.071	**0.972**	**0.563**
**UFRGS-PV-0848-T**	**2.543**	**2.000**	**1.971**	**0.195**	**0.143**	**0.165**	**1.200**	**0.880**	**0.286**	**0.143**	**0.100**	**0.686**	0.645
**UFRGS-PV-0972-T**	**1.930**	**1.430**	**1.611**	**0.220**	0.144	0.203	**1.095**	**0.630**	**0.223**	**0.148**	**0.130**	0.716	**0.373**
**UFRGS-PV-1152-T**	**2.459**	**2.297**	**2.243**	**0.203**	0.146	0.263	**1.050**	0.751	0.342	0.206	0.061	0.888	**0.633**
**UFRGS-PV-1153-T**	2.545	**1.703**	1.966	**0.330**	**0.222**	**0.340**	**1.140**	**0.743**	**0.445**	**0.204**	**0.111**	0.812	**0.835**

I) lower jaw length; II) dentary length; III) length from the most anterior and ventral point of secondary bone to mandibular fossa; IV) anterior height of dentary; V)anterior height of secondary bone; VI) height of dentary in the inflexion point; VII) length from the anterior portion of dentary to anterior portion of the coronoid process; VIII) length from the anterior portion of the dentary to the posterior portion of last additional tooth. IX) length from the anterior portion of last additional tooth to anterior portion of coronoid process; X)length of base of last additional tooth; XI) height of last additional tooth; XII) length from the coronoid process; XIII) height of coronoid process. The “real” measurements are represented in bold type.

A survey for the taphonomic signatures was performed as well as the establishment of the degree of tooth wear, which is related with ontogeny in the group of Clevosaurs [20]. These data were condensed into a Table 3.

**Table 3:** Taphonomic signatures and degree teeth wear.

Especimen	A	B1	B2	E	Wear of the Teeth
**MCN-PV-2852**	3	2	1	1	2
**UFRGS-PV-0606-T**	4	0	3	0	3
**UFRGS-PV-0613-T**	1	2	1.5	1	0
**UFRGS-PV-0735-T**	2	2	1.5	1	3
**UFRGS-PV-0738-T**	4	0	1	1	3
**UFRGS-PV-0739-T**	4	0	2	2	2
**UFRGS-PV-0748-T**	2	5	0	3	2.5
**UFRGS-PV-0750-T**	4	0	2	2	2
**UFRGS-PV-0752-T**	2	2	0.5	1	2.75
**UFRGS-PV-0753-T**	2	2	0	4	?
**UFRGS-PV-0754-T**	3	2	1.5	2.5	2
**UFRGS-PV-0758-T**	4	1.5	1	0.5	2.25
**UFRGS-PV-0974-T**	2	2	0	0.5	1.75
**UFRGS-PV-0848-T**	3	4.5	1	0	1
**UFRGS-PV-0972-T**	2	3	1	3	1
**UFRGS-PV-1152-T**	2	0.5	0	1.5	?
**UFRGS-PV-1153-T**	4	0	1	0.5	3

A: degree of articulation; 4 = skull and lower jaw articulated with the vertebrae; 3 = skull articulated with the lower jaw, 2 = dentary articulated with the other bones of the lower jaw (articular complex); 1 = isolated dentary; B1: degree of fragmentation with the presence of fragments in the sample (only for dentary), zero is equal to absence of fragmentation, the numbers 1 to 5 represent the total of fragments, but when the size of the fragment is very small it is counted as 0.5; B2: fragmentation degree, in the absence of fragments in the sample (only for dentary), the number represents the total of fractures that represent absent fragments, but when the size of the fragment is very small it is counted as 0.5; E: level of fractures: 0 = absent; 0.5 = one weak fracture; 1 = when fractures are very weak and few; 1.5 = when fractures are very weak and few, and one fracture is stronger; 2 = when fractures are weak (more than five, and get counting) or/and few stronger fractures; 2.5 = when fractures are weak (more than five, and get counting) or/and few stronger fractures (no more than five); 3 = when fractures are strong and more than 5, and/or many weak fractures; 4 = many and stronger fractures. These observations were made with stereoscopic microscope. Teeth wear in occlusal surface, is from 0 to 3; where 0 means no worn and 3, very worn.

#### Missing data

After linear measurements were obtained, this information was used to estimate the missing values through the method “*Bayesiam PCA missing value estimator*” by the function “bpca” of the package “pcaMethods” [54, 55, 56] of the software R vs. 3.0.0 [57]. This method was originally created by [54] for estimated missing data in array of DNA. It consists of three elementary processes: a principal component (PC) regression; Bayesian estimation and; expectation-maximization (EM) [54]. It is a highly complex method requiring multiple iterative matrix inversions and attempts to replace the missing value based via PCA by regressing the remaining values against the principal components for complete values [58]. It has been used for missing data estimation in morphometrics [7], and according to Brown *et al*. [58], this method is recommendable because introducing the least amount of error when handling with missing data. Then, the same procedure was done with the method “*correlated variable regression”* using the function “best.reg” of the package “*Lost”* [59] of the software R [57]. This method used the variable most highly correlated with the variable experiencing missing data [58].

Such estimation was repeated three times for each method. It was given priority to the first method because it is the most accurate [58], only in the case when the estimated value were incoherent, we used the estimates value thrown out by the second method. With the help of new measures, we performed reconstructions of the broken specimens and their location of the missing landmarks.

### Geometric morphometrics

**Fig 7:**
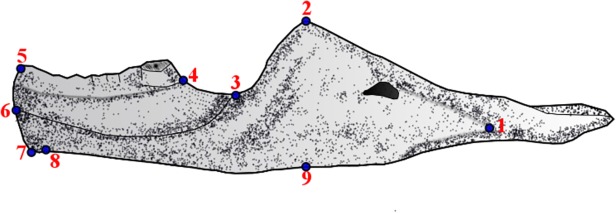
Landmarks from 1 to 9.

For the geometric morphometric analyses 9 landmarks were employed (Fig. 7 and Table 4). These landmarks were not influenced by the available view of each dentary (lateral or medial). Zeldicht *et al*. [60] recommended the optimal number of landmarks to be a third of the *n* size of the sample, however, while reviewing the literature, it was observed that some authors do not agree with this proportion, such as Jones [2](*n* = 49 using 54 landmarks), Campione and Evans [7] (*n* = 17 and use 13 landmarks) and Meloro and Jones [12] (*n* = 39 and using 54 landmarks), Drake and Klingenberg [61] (*n* = 47 and using 64 landmarks).

**Table 4:** Landmarks used.

Landmark	Definition
1	Suture between of dentary, angular and surangular
2	The most dorsal point of coronid process
3	Inflexion point on the anterior margin of coronid process
4	The most posterior point of the most posterior teeth
5	The anteior and superior point of dentary
6	The most antero-ventral point of secondary bone
7	The most antero-ventral point of dentary
8	Ventral inflexion point of dentarý
9	The ventral point, result of a vertical line drawn from the landmark 2 to the ventral edge of dentary

**Fig 8:**
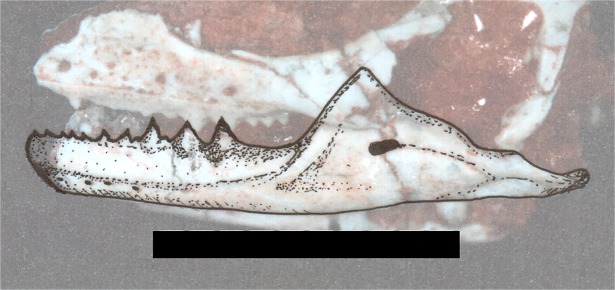
Example of combination of the photos plus the reconstructions. Specimen UFRGS-PV-0613-T.

The landmarks were placed directly on the combination of the photos plus the reconstructions (Fig. 8) using the software TpsDig2 [62]. Using the package “*Geomorph*” [63] of the software R vs 3.0.0 [57] it was performed as General Procrustes Analysis (using the function “gpagen”) for removing non-shape variation (position, size and orientation) and to align specimens to a common coordinate system [60, 63, 64]. A Principal Analysis Components (PCA) was performed in the function “plotTangentSpace” to identify the main components of shape variation within the sample Thin-plate spline transformations were generated to help demonstrate the difference between the centre of morphospace (the mean shape) and other areas of morphospace e.g. along principle component axes [63, 64]. The geometric morphometric analyses were repeated twice with the aim of decreasing the chances of errors.

The principal component scores were provided by “*Geomorph*” twice and were compared among them and then with: (1) the taphonomic signatures: for this comparison, we created new scores of taphonomic signatures (AF) using PCA, so that the values were independent of each other; and (2) the degree of wear of the teeth (WT), feature that was used as a proxy of age. These comparisons were made using a Pearson’s test correlation with the software PASW v.18 [65].

One regression analysis was performed with the Centroid Size as the independent variable, and PC1 as dependent variable, because PC1 against CS is often used in an ontogenetic sample measuring allometry, once the PC1 is usually the allometric shape component [66].

Finally, seven new analyses were performed but with some modifications in the size of “N” using the specimens with less “missing data”; and also without landmark 2 and 9. This was done to detect any possible effect of the incomplete specimens or of these two landmarks (S1 Text). Because these new results have not modified the original ones, only the results of the analyses whit 10 most complete dentary (whit less missing data: MCN-PV-2852; UFRGS-PV-0606-T; UFRGS-PV-0752-T; UFRGS-PV-0753-T; UFRGS-PV-0754-T; UFRGS-PV-0848-T; UFRGS-PV-0972-T; UFRGS-PV-1153-T; UFRGS-PV-0613-T; UFRGS-PV-0748-T) and 7 landmarks (without landmark 2 and 9), are discussed. However, the respective tables and figures of all additional analyses are presented as supporting material (S1–S15 Tables, S1 Text, and S1 Fig.).

## Results

In the PCA, the most explicative variation was displayed by PC1 (83.3%) and PC2 (10.0%), representing 93.3% of the total variation (Table 5).

**Table 5:** Importance of components (without LM2 and LM9 and N = 10).

	Standard deviation	Proportion of variance	Cumulative proportion
**PC1**	0.010	0.833	0.833
**PC2**	0.035	0.100	0.933
**PC3**	0.022	0.042	0.975
**PC4**	0.012	0.013	0.987
**PC5**	0.010	0.008	0.996
**PC6**	0.006	0.003	0.998
**PC7**	0.004	0.001	0.999
**PC8**	0.003	0.001	1.000
**PC9**	0.001	0.000	1.000
**PC10**	0.000	0.000	1.000

Importance of components of the analysis whit ten specimens and without landmark 2 and landmark 9.

**Fig 9:**
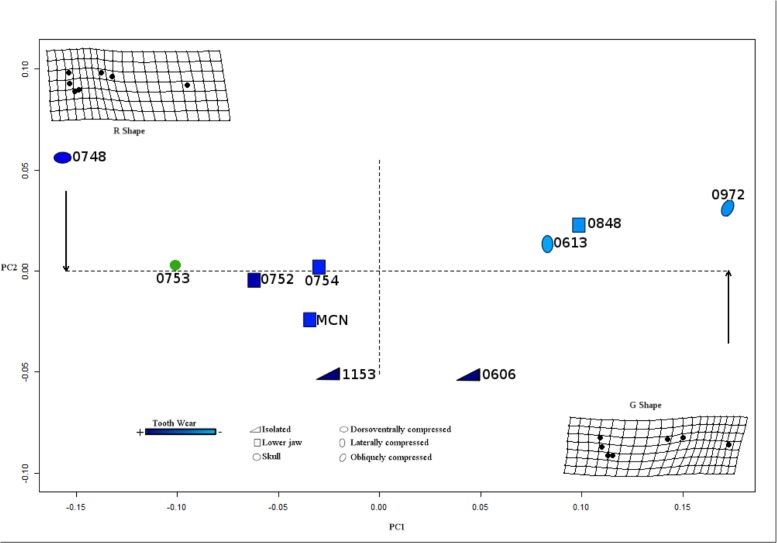
Plot PCA. With thin plate of the analysis whit ten specimens and without landmark 2 and landmark 9. Note: in green color are the specimens without tooth information.

It can be observed (axis X) in the resulting plot for the PC1 versus PC2 in the tangent space, together with the visualization of the deformation grids for shapes along PC1 (Fig. 9), that as the dentary grows, the absolute value of PC1 also grows, but in a negative way. In the deformation grids we can also see that the “G Shape” is located in positive value in the axis X, and the “R shape” is located in the negative value for the axis X, and the difference between these shapes are: as the PC1 value moves to the left (negative values) in the plot, the proportion (rate) of the total length of the dentary relative to the tooth row portion (RD), decreases. On the other hand, the correlation between PC1 and the teeth wear was r = −0.640, also a strong correlations. But the correlations between PC1 and the scores of taphonomic signatures are weak (Table 6).

**Table 6:** Pearson correlation (without LM2 and LM9 and N = 10).

	WT		DM		AF1		AF2		AF3	
	r	p	r	p	r	p	r	p	r	p
**PC1**	**−0.640**	0.064	**0.715**	**0.020**	0.295	0.408	−0.340	0.337	0.486	0.154
**PC2**	−0.522	0.149	0.014	0.968	**−0.876**	**0.001**	0.073	0.841	0.437	0.206
**PC3**	0.222	0.565	0.417	0.230	0.055	0.880	−0.701	0.687	−0.303	0.396

Values for Person Correlation whit ten specimens and without landmark 2 and landmark 9.r and p values. Stronger correlations are highlighted in bold type.

In relation to PC2, as shown in the plot (Fig. 9), the trend is that dorsoventrally compressed skulls are located in the highest positive values for PC2 (axis Y); whereas the ones closer to zero of that axis are the laterally compressed skulls; and generally, below zero (higher and negatives in Y), isolated lower jaws appears, and when their value increases, the specimens are more incomplete (the patterns are inverted, like mirror image, in the others analysis see S1 Fig.). In contrast with the results of the Pearson’s test between PC1 versus the teeth wear and the taphonomic signatures (AF), the correlation of the PC2 versus teeth wear (WT) is very weak; but when PC2 is compared with the scores 1 for taphonomic signatures, the result is stronger, with a value in the first analysis of r = 0.876 (Table 6).

**Fig 10:**
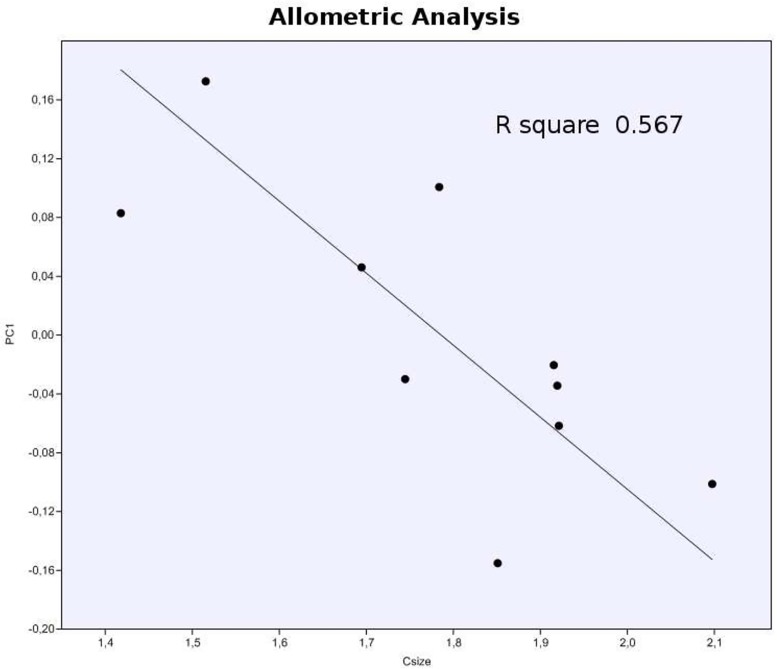
Allometric Analysis (PC1 versus Centroid Size). Formula (A+ BX): 0.659−0.369(CS); R = −0.753; R square 0.567; P = 0.012.

In the allometric analyses, in the first regression (CS versus PC1) the result was p = 0.012, and this is statistically signifcant.But with the second regression (CS versus PC2), the result was not significant (Fig. 10).

It was detected that our sample comprises only juvenile and adult specimens, with absence of earlier ontogenetic stages, from T to T4 of Robinson [17] (these ontogenetic stages range from few weeks to few months). This asseveration is given as: 1) in all juvenile specimens are present additional and hatchling teeth; hatchling teeth alone occur exclusively in the hatchling individuals [20], and 2); in all specimens it is present a diastema between the last additional tooth of the dentary and the coronoid process; usually, this diastema is absent in hatchling *Sphenodon*, but in mature specimens, it is produced one diastema between the last additional tooth and the coronoid process [17].

Thus, the specimen UFRGS-PV-0848-T is recognized as a juvenile, and it is confirmed that UFRGS-PV-0613-T and UFRGS-PV-0972-T are also juveniles as proposed by Bonaparte and Sues [26] and Arantes (unpublished data). The specimen UFRGS-PV-0974-T is recognized as a young adult, and the remaining specimens of the sample are recognized as mature forms.

## Discussion

Principal Component 1 (83.3%) is significantly associated with differences in tooth wear and describes differences in the proportions of the coronoid process of the tooth row length relative to jaw length. Principal Component 2 (10.0%) is significantly associated with differences in taphonomic distortions.

Morphological differences are consistent with changes that might occur during ontogeny. However, the ontogenetic changes are not the only cause for these differences, once other processes related to the structure of population, such as sexual dimorphism, phenotypic variations of individuals, and taphonomic biases could help to explain the size variations inside the sample.

### Ontogenetic implications

Based on the results of PC1, the deformation in the thin plate (Fig. 9 and S1 Fig.), and our observations on the dentition, a model of ontogenetic development was inferred.

#### Dentition

In juveniles specimens the hatchling teeth of the dentary are exposed (e.g. Fig. 4F), and in general they are not worn in the occlusal surface (e.g Fig. 4: F, G, H). These teeth show alternation in size which is a characteristic of the rhynchocephalia [19]. It is possible to see the decrease of their number from juveniles to mature specimens, which is related to the secondary bone growth, as observed in other *Clevosaurus* [20]. Three or four little additional teeth are placed in the most posterior part of the maxilla (e.g. Fig. 3D; 9: F, G). Strong score marks are observed in the lateral surface of the adding teeth of the dentary (e.g. Fig. 4: F, G, H). (Table 1)

In the mature specimens we can see high wear in the occlusal surface of the crown of the dentary teeth, and the lateral score marks are poorly visible or even absent (e.g. Fig. 4: A, B, C, D, E, H). Probably they have been enclosed by the secondary bone.

These different patterns of wear in the teeth can be possibly correlated with the differences in the way of type food between adults and juveniles, as previously proposed for *Clevosaurus* by Fraser [20] and Fraser and Walkden [35], and also occurring in *Sphenodon* [2, 17,67, 68]. According to these authors, the juveniles were insectivores, whereas the mature specimens were omnivores, but *Clevosaurs* feeding habits are still in discussion from facultative herbivory to carnivory [12, 30, 35, 53]. In the particular case of *C*. *brasilieinsis*, it is supported the hypothesis that juveniles have been restricted to an insectivorous diet, as proposed for the juveniles of all Triassic rhynchocephalian, since the features of their dentition are well adapted for trapping small insects [35]. It has been suggested that the adult Clevosaurs and other taxa with six distinct rows of palatal teeth also possessed strong rounded snouts and robust palatal bones, and the palatal teeth themselves in these taxa are stout and suggestive of a triturating surface [2]. Added to this, we observed in *C*. *brasiliensis* an apparent tendency to develop a greater amount of secondary bone, and consequently a greater number of teeth hidden by it than other *Clevosaurus*. In order to check this observation, some additional measurements would have to be made but the available published data on *Clevosaurus* do not allow this. This greater amount of secondary bone produces a sharp cutting edge in the anterior region [2, 35], larger than in other known species of *Clevosaurus*.

As aforementioned, the mature individuals of *C*. *brasiliensis* do not exhibit strong score marks in the lateral teeth surface, as in other mature individuals of the same genus, as, for example, *C*. *hudsoni* (pers. obs. PRM). It is possible that mature individuals of *C*. *brasiliensis* had more interaction between additional teeth of the dentary with the “groove” of palatine and palatine teeth, than juveniles and even mature individuals of other *Clevosaurus*. These features are probably indicative of an omnivorous diet for *C*. *brasiliensis*, different from what was proposed for *C*. *hudsoni*, which would be mainly carnivorous according to Jones [2, 36] and Apesteguía (unpublished data).

#### Dentary growth

**Fig 11:**
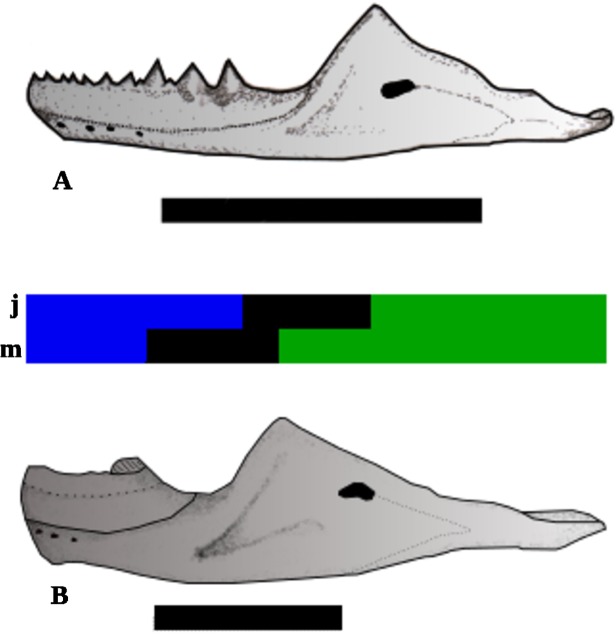
Reconstruction of the dentary growth. **A)** Juvenile individual; **B**) Mature individual. The tricolor, bar represents the regions of dentary: Blue = the tooth row; Black = diastema; Green = the posterior region; j = the regions in juvenile individual, and m = regions in mature individual Scale bars 1 cm.

In *Sphenodon*, “the dentary increases in length posteriorly, and may continue to do so after the implantation of the last member of the additional series, producing an diastema between the last additional tooth and the coronoid process of the dentary” [17], and the same was inferred for *Cynosphenodon huizachalensis* [34]. In the thin plate (Fig. 9, and S1 Fig.), we see that the dentary is gracile in the “G shape” (juveniles), and that in the “R shape” (adults) the dentary is more robust. Also, the tooth row is relatively longer in juvenile compared to mature specimens (Fig. 11). Because in *Clevosaurus* there are no successional teeth, the only reference point are the last additional teeth, and it is not possible to compare the other points of growth in the anterior part with *Sphenodon* and *Cynosphenodon huizachalensis*.

The rate of dentary growth observed along the thin plate (Figs. 9, 11 and S1 Fig.) is congruent with the result of the allometric analysis within the PC1 with Centroid size (CS), whose value of p (0.012) was significant (Fig. 10). It is noteworthy that, these methods (allometric analysis and Thin plate), provide complementary visualizations of the allometric and ontogenetic patterns [66]. The result of the regression of value for R square is 0.567, which statistically means that 56.7% of the variations of PC1 can be explained by allometric process during ontogeny as observed in the Thin Plate. This would be reflected in the difference of the growth rate of the posterior portion of the dentary with respect to its anterior portion.

The number of teeth is associated with the skull shape in Rhynchocephalia [2]. When a taxon has a smaller number of teeth, like *Sphenodon* and *Clevosaurus*, the tendency is to possess a skull more robust skull with greater room for the adductor muscles [2]. Thus, fewer teeth mean a smaller surface area of initial contact with food items, and greater loading from bite forces [2, 12, 36]. In the ontogeny of *Clevosaurus*, the tendency is to reduce the number of teeth. We also observed another difference between dentaries of juveniles and mature specimens. The former exhibit gracile dentaries, whereas the latter robust ones, which could support the idea of differences in feeding habits among juveniles and mature specimens.

The evolution of Clevosaurs is characterized by a shortening of the snout, so that the nares become more upright, and elongation of the posterior jugal process [12]. Therefore, it is possible that this trend is reflected in the ontogenetic development of *Clevosaurus*. Particularly, in *C*. *brasiliensis*, it was observed an allometric growth with respect to the anterior and posterior portion of the dentary, wherein the posterior portion has a higher growth. This allometric growth, together with the development of a more robust dentary, is probably accompanied by the allometric growth of the skull, similar to the trend exhibited by the group.

### Sexual dimorphism

In the analysed sample there are specimens with the same shape, but that display different length (e.g. MCN-PV-2852, UFRGS-PV-0750-T, UFRGS-PV-0752-T, UFRGS-PV-0754-T, UFRGS-PV-0758-T, see S1 Fig.). This may be due to sexual dimorphism, as it is well known in Lepidosauria. In the case of lizards, sexual dimorphism is common with respect to the head size, and associated with bite force [69]. These also occur in the majority of Squamata and in *Sphenodon* [70], in which the head is typically male-biased [68, 69, 70]. In both cases, this can be related with different food sources, and with male-male interactions, because a stronger bite force is advantageous in acquiring and maintaining territories, being determinant for fitness in male *Sphenodon* [68, 70].

In addition to these, the different degrees of teeth wear, besides related with the ontogenetic stage, also may be related with sexual dimorphism (stronger bite force in males) and therefore with the foraging habits. Finally, part of the wearing of the teeth may be product of taphonomic processes, as discussed in the following section.

### Taphonomic implications

As it has already been described, the PC2 represents 10.0% of the variation of the form, and this is influenced by taphonomic processes. The deformation produced by taphonomic biases can result in morphological interpretations that could lead to the equivocal creation of two or more species. In fact, there are studies using geometric morphometrics that have succeeded in elucidating deformation patterns caused by taphonomic process, and they have detected taxa in synonymy (i.e. Hedrick and Dodson [8]). In our monotypic sample, it is only possible to observe some diagenetic influence on the morphology, reflected in deformed skulls (see [71]). In the plot (Fig. 8) the trend is shown between the PC2 and the type of compressed skull, so that the dorsoventrally compressed skulls are located in the highest positives values, and the laterally compressed skulls are located closer to the zero. Unfortunately, the number of skulls in the sample is not enough to confirm the hypothesis that one of the processes that had a substantial influence on the shape variation was the fossil diagenesis.

Regarding biostratinomy, we find a stronger correlation within the taphonomic signatures and the PC2 especially for disarticulation (A) and degree of fragmentation (B1 and B2). These signatures were most influenced in the AF1, the score that had the higher correlation with the PC2 (Table 6). Finally, we do not rule out the possibility that the PC2 can be related to a lack of data, since the signatures are related to loss of structures and therefore this may reflect some degree of error due to the *missing data* and the reconstructions.

Concerning the temporal resolution, the time-averaging involved in the generation of fluvial and deltaic environments generally takes from 10^1^ to 10^4^ yrs [72, 73]. So it is very likely that the bed containing *C*. *brasiliensis* can encompass such temporal resolution, implying time enough for the succession of one or more populations, and consequently the development of the small morphological differences. Thus, it is important to remember that usually the most recent populations are better represented in number of individuals [72], this could have occurred in our sample. Unfortunately, given the nature of the rock where the fossils are included, a massive sandstone, it was not possible to obtain a finer stratigraphic control for a more accurately time-resolution. Finally, regarding the structure of the population, it is probable that the thanatocoenosis is bimodal, generated by selective death. However, there is a bias towards the mature individuals. This may be due to the fact that generally immature organisms are weaker and less ossified, and therefore, they possess smaller chances of preservation.

## Conclusions

The morphological differences among the specimens of the sample can be assigned to characteristics that are often influenced by changes during the ontogenetic development, like tooth pattern and wear, dentary growth and secondary bone growth. In *C*. *brasiliensis* the lateral wear is stronger in the juvenile specimens, being even absent in some mature specimens. In contrast, the wear in the occlusal surface is stronger in the mature specimens and smooth or absent in the juveniles ones. The dentary in juveniles is slender, bearing more hatchling teeth and the dentary carrier portion of teeth with respect to its length is greater than in mature individuals. In the latter, the dentary is more robust and the hatchling teeth are enclosing by the secondary bone.

We believe that there are differences in the way of processing food between adults and juveniles, being juveniles restricted to an insectivorous diet [20,35]. We hold that mature specimens of *C*. *brasiliensis* have an omnivorous habit, and it is probable that they have consumed more plant items, compared with to other *Clevosaurus*. This is supported by the fact that *C*. *brasiliensis* has a greater development of the secondary bone, which creates a bigger edentulate portion of the beak-shaped dentary in comparison with other *Clevosaurus*, and that their teeth do not resemble the mammalian carnassials as in *Clevosaurus hudsoni* [2,12]. This edentuale portion is similar to the “beak” of *Uromastix* lizard and it is used for cropping plant [17, 35]. The habit of herbivoy was previously proposed for the rhynchocephalian *Sigmala Sigmala*, *Pelecymala robusta*, *Sphenotitan* as well as the facultative herbivory for *Clevosaurus hudsoni*, due to the presence of a “beak”[19, 20, 27, 28, 35]. Finally the group that was distinguished for having successflly explored herbivory was the Eilenodontines of America (Late Jurassic to Late Cretaceous) [25, and Apesteguía (unpublished data)].

In the case of rhynchocephalians, some important diagnostic features change during the development, such as the proportion of the orbit with respect to the skull size [2], besides the other teatures discussed above. The geometric morphometrics is an effective tool for showing these kinds of biases and for elucidating the shape variation as well as its causes. In the specific case of *Clevosaurus brasiliensis*, due to the absence of caniniform teeth, which could help to draw reference lines to detect the dentary growth (as in *Sphenodon* and *Cynosphenodon huizachalensis*), the geometric morphometry was an alternative tool that helped to understand the dentary growth pattern.

## Supporting Information

S1 Fig Plot PCA with thin Plate of the all specimens. Note in green color are the specimens without tooth information.
(TIFF)

S1 Table Importance of components (all specimens). (XLS)

S2 Table Pearson Correlation (all specimens). (XLS)

S3 Table Importance of components (without UFRGS-PV-0972-T). (XLS)

S4 Table Pearson Correlation (without UFRGS-PV-0972-T). (XLS)

S5 Table Importance of components (N = 10). (XLS)

S6 Table Pearson Correlation (N = 10). (XLS)

S7 Table Importance of components (N = 8). (XLS)

S8 Table Pearson Correlation (N = 8). (XLS)

S9 Table Importance of components (without LM 2 and LM 9). (XLS)

S10 Table Pearson Correlation (without LM 2 and LM 9). (XLS)

S11 Table Importance of components (without LM 2 and LM 9 and UFRGS-PV-0972-T). (XLS)

S12 Table Pearson Correlation (without LM 2 and LM 9 and UFRGS-PV-0972-T. (XLS)

S13 Table Importance of components (without LM 2 and LM 9 and N = 8). (XLS)

S14 Table Pearson Correlation (without LM 2 and LM 9 and N = 8). (XLS)

S15 Table Allometric Analysis. (XLS)

S1 Text Supporting Information. (DOC)
