# Supplementary figures and images for: Dentary Morphological Variation in Clevosaurus brasiliensis (Rhynchocephalia, Clevosauridae) from the Upper Triassic of Rio Grande do Sul, Brazil
Source: PLoS One. 2015 Mar 20;10(3):e0119307. doi: 10.1371/journal.pone.0119307 (PMC4368672; doi:10.1371/journal.pone.0119307)

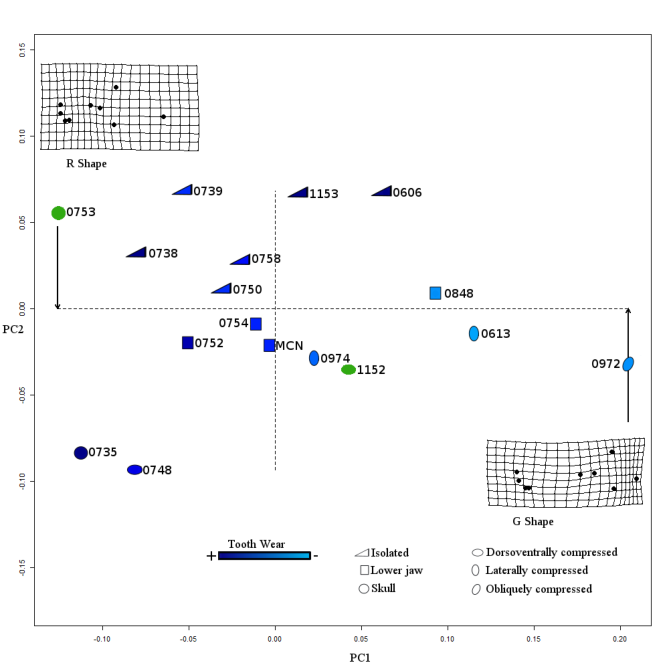

Supplement: S1 Fig — Note in green color are the specimens without tooth information. (TIFF) [file pone.0119307.s001.TIFF]
