# Supplementary material for: Dentary Morphological Variation in Clevosaurus brasiliensis (Rhynchocephalia, Clevosauridae) from the Upper Triassic of Rio Grande do Sul, Brazil
Source: PLoS One. 2015 Mar 20;10(3):e0119307. doi: 10.1371/journal.pone.0119307 (PMC4368672; doi:10.1371/journal.pone.0119307)
Supplement: S1 Text — (DOC) [file pone.0119307.s017.doc]

**Supporting Information**

The original analyses correspond to S1- 2 Tables and S1 Fig.

Three new analyses were performed with different size of “n”. 1) Without de specimen UFRGS-PV-0972-T, because it is the only one obliquely compressed (S3 and S4 Tables); 2) “n” = 10 with the specimens MCN-PV-2852, UFRGS-PV-0606-T, UFRGS-PV-0613 T, UFRGS-PV-0748-T, UFRGS-PV-0752-T, UFRGS-PV-0753-T, UFRGS-PV-0754-T, UFRGS-PV-0848-T, UFRGS-PV-0972-T, UFRGS-PV-1153-T (S5 and S6 Tables); and “n” = 8 with the specimens MCN-PV-2852, UFRGS-PV-0606-T, UFRGS-PV-0752-T, UFRGS-PV-0753-T, UFRGS-PV-0754-T, UFRGS-PV-0848-T, UFRGS-PV-0972-T, UFRGS-PV-1153-T (S7 and S8 Tables).

Subsequently, the landmarks 2 and 9 were removed, because they are co-dependent landmarks, and the previous four analyses were repeated (S9-14 Tables, and Table 5 and 6). Finally in the S15 Table are exhibited the results of the allometric analysis for each of the new combination of “n” size.

In general, in all cases the observed pattern with regard to the proportions in the teeth is the same that the pattern described in the text. The results of the correlation are very similar with the original result, but in particular case of the dentition wear, the values range between the statistically significant or close to this.
